# Biochemical Characterization and Pharmacological Properties of New Basic PLA_**2**_ BrTX-I Isolated from *Bothrops roedingeri* (Roedinger's Lancehead) Mertens, 1942, Snake Venom

**DOI:** 10.1155/2013/591470

**Published:** 2012-12-30

**Authors:** Mauricio Aurelio Gomes Heleno, Paulo Aparecido Baldasso, Luis Alberto Ponce-Soto, Sérgio Marangoni

**Affiliations:** Department of Biochemistry, Institute of Biology, State University of Campinas (UNICAMP), P.O. Box 6109, 13083-970 Campinas, SP, Brazil

## Abstract

BrTX-I, a PLA_2_, was purified from *Bothrops roedingeri* venom after only one chromatographic step using reverse-phase HPLC on **μ**-Bondapak C-18 column. A molecular mass of 14358.69 Da was determined by MALDI-TOF mass spectrometry. Amino acid analysis showed a high content of hydrophobic and basic amino acids as well as 14 half-cysteine residues. The total amino acid sequence was obtained using SwissProt database and showed high amino acid sequence identity with other PLA_2_ from snake venom. The amino acid composition showed that BrTX-I has a high content of Lys, Tyr, Gly, Pro, and 14 half-Cys residues, typical of a basic PLA_2_. BrTX-I presented PLA_2_ activity and showed a minimum sigmoidal behavior, reaching its maximal activity at pH 8.0, 35–45°C, and required Ca^2+^. *In vitro*, the whole venom and BrTX-I caused a neuromuscular blockade in biventer cervicis preparations in a similar way to other *Bothrops* species. BrTX-I induced myonecrosis and oedema-forming activity analyzed through injection of the purified BrTX-I in mice. Since BrTX-I exerts a strong proinflammatory effect, the enzymatic phospholipid hydrolysis might be relevant for these phenomena; incrementing levels of IL-1, IL-6, and TNF**α** were observed at 15 min, 30 min, one, two, and six hours postinjection, respectively.

## 1. Introduction

PLA_2_s (phosphatide 2-acylhydrolase, EC 3.1.14) represent a superfamily of lipolytic enzymes which specifically catalyze the hydrolysis of the ester bond at the sn-2 position of glycerophospholipids resulting in the generation of fatty acid (arachidonate) and lysophospholipids. The PLA_2_ superfamily consists of about 15 groups which are further subdivided into several subgroups, all of which display differences in terms of their structural and functional specificities. However, the four main types or classes of PLA_2_s are the secreted, the cytosolic, the Ca^2+^-independent and the lipoprotein-associated PLA_2_ [1], PLA_2_ structure/function, mechanism, and signaling [2].

Snake venom PLA_2_s displays a variety of activities, such as neurotoxicity, myotoxicity, cardiotoxicity, and hemolysis that may be modulated by specific receptors located on target cells [3–6]. Indeed, PLA_2_ receptors classified as kinds M and N [7] have been identified in various kinds of cells, including vascular smooth muscle cells, platelets, neutrophils, chondrocytes, fibroblasts, hepatocytes, and mesangial cells, as well as in brain, lung, and skeletal muscle [8, 9]. Snake venom PLA_2_ can bind to M receptors, which are the most common kind found in human macrophages and muscle cells, and these may mediate some of the deleterious actions of venom PLA_2_s, although that was not conclusively demonstrated [5, 6].

Peru has a rich and diverse herpetofauna that includes venomous snake species of the families Elapidae (16 species of *Micrurus *and the pelagic sea snake *Pelamis platurus*) and Viperidae (15 species) [10]. Snakebite envenomations represent a public health problem in this country. The vast majority of snakebites in Peru are inflicted by species of the genus *Bothrops *(familyViperidae) [11]. *Bothrops atrox*, *Bothrops brazili*, and* Bothrops bilineatus *are distributed in the tropical rainforests located in the eastern part of the country, whereas *Bothrops barnetti *and *Bothrops roendingeri *are found in the western dry coastal regions [10–12].

This variety of pharmacological roles derives from an accelerated microevolutionary process through which a high rate of amino acid substitutions have occurred in molecular regions located mainly at the surface of these molecules [13–15]. The purpose of this paper is to isolate, biochemically and pharmacologically characterize a basic PLA_2_ from* Bothrops roedingeri* venom, BrTX-I.

## 2. Materials and Methods

### 2.1. Venom and Reagents

The venom was obtained from the adult specimens of *Bothrops roedingeri* captured in the vicinity of Arequipa-Perú. Swiss mice (18–20 g) were supplied by the Animal Services Unit of the State University of Campinas (UNICAMP). All experiments were conduced in accordance with guidelines of the Committee for Ethics in Animal Research, UNICAMP No. 2006-1 (Campinas-Brazil). The reagents used in this work were of analytical or sequencing grade.

### 2.2. PLA_2_ Activity

PLA_2_ activity was measured using the assay described in [16, 17], modified for 96-well plates [18]. The standard assay mixture contained 200 *μ*L of buffer (10 mM Tris-HCl, 10 mM CaCl_2_, 100 mM NaCl, pH 8.0), 20 *μ*L of substrate (4-nitro-3-octanoyloxy-benzoic acid), 20 *μ*L of water, and 20 *μ*L of PLA_2_ in a final volume of 260 *μ*L. After the addition of PLA_2_ (20 *μ*g), the mixture was incubated for up to 40 min at 37°C, with the absorbance being read at 10 min intervals. The enzyme activity, expressed as the initial velocity of the reaction (*Vo*), was calculated based on the increase in absorbance after 20 min.

All assays were done three times and the absorbances at 425 nm were measured using a VersaMax 190 multiwell plate reader (Molecular Devices, Sunnyvale, CA, USA).

### 2.3. Reversed-Phase HPLC (RP-HPLC)

Five milligrams of the venom was dissolved in 200 *μ*L solvent A (TFA 0.1%, pH 3.5). The resulting solution was clarified by centrifugation and the supernatant was applied to a *μ*-Bondapak C18 column (0.78 × 30 cm; Waters 991-PDA system). Fractions were eluted using a linear gradient (0–100%, v/v) of acetonitrile (solvent B) at a constant flow rate of 1.0 mL/min over 40 min. The elution profile was monitored at 280 nm, and the collected fractions were lyophilized and conserved at −20°C.

### 2.4. Electrophoresis SDS-PAGE

The relative molecular mass of the protein was determined by SDS-PAGE [19]. The molecular mass markers were (in kDa): phospholipase B—94, albumin—67, ovalbumin—43, carbonic anhydrase—30, soybean trypsin inhibitor—20, and lysozyme—14.

### 2.5. Amino Acid Analysis

Amino acid analysis was done on a Pico-Tag amino acid analyzer (Waters Corporation, Massachusetts, USA) as described by [20]. The purified protein (30 *μ*g) was hydrolyzed at 105°C for 24 h in 6 M HCl acid (Pierce sequencing grade) containing 1% phenol (w/v). The hydrolyzates were reacted with 20 *μ*L of derivatization solution (ethanol : triethylamine : water : phenylisothiocyanate, 7 : 1 : 1 : 1, v/v) for 1 h at room temperature after the phenylthiohydantoin (PTC)-amino acids were identified and quantified by HPLC by the comparison of their retention times and peak areas with those of a standard amino acid mixture.

### 2.6. Reduction and Alkylation

Purified lyophilized protein from RP-HPLC was resuspended in 8 M urea containing 10 mM DTT at pH 8.0 and the disulfide bridges were then reduced by incubation at 37°C for 2 h. Since the number of cysteine residues in the protein was initially unknown, the optimum concentration of iodoacetamide for alkylating the free thiols was derived empirically, based on results obtained from incubations using various concentrations of iodoacetamide and different amounts of protein, with each mixture being analyzed by mass spectrometry [21]. Based on these preliminary experiments, a 30% molar excess of iodoacetamide relative to the total number of thiols was eventually chosen and the mixture was incubated for 1.5 h at 37°C in the dark. The reaction was ceased by injecting the mixture onto a RP-HPLC column followed by lyophilization of the collected peak.

### 2.7. Enzymatic Hydrolysis

The purified proteins were hydrolyzed with sequencing grade bovine pancreatic trypsin in 0.4% ammonium bicarbonate, pH 8.5, for 4 h at 37°C, at an enzyme : substrate ratio of 1 : 100 (w/w). The reaction was ceased by lyophilization.

### 2.8. Mass Spectrometry

All mass spectra were acquired using a quadrupole-time of flight (Q-TOF) hybrid mass spectrometer Q-TOF Ultima from Micromass (Manchester, UK) equipped with a nano Zspray source operating in a positive ion mode. The ionization conditions of usage included a capillary voltage of 2.3 kV, a cone voltage and RF1 lens of 30 V and 100 V, respectively, and a collision energy of 10 V. The source temperature was 70°C and the cone gas was N_2_ at a flow of 80 L/h; nebulizing gas was not used to obtain the sprays. Argon was used for collisional cooling and for fragmentation of ions in the collision cell. External calibration with sodium iodide was made over a mass range from 50 to 3000 *m/z*. All spectra were acquired with the TOF analyzer in “Vmode” (TOF kV = 9.1) and the MCP voltage set at 2150 V.

### 2.9. Analysis of Native and Alkylated Protein

Lyophilised RP-HPLC fractions of intact native and alkylated protein were dissolved in 10% acetonitrile in 0.1% TFA and was introduced into the mass spectrometer source with a syringe pump at a flow rate of 500 nL/min. Mass spectra were acquired over the mass range of 1000–2800 *m/z* for the native protein and over the range of 800–2000 *m/z* for the alkylated protein, both at a scan speed of 1 s/scan. The masses were analyzed by the MassLynx-MaxEnt 1 deconvolution algorithm. The data obtained were processed using the Mascot MS/MS Ion Search software http://www.matrixscience.com/.

### 2.10. De Novo Sequencing of Tryptic Peptides

Alkylated tryptic peptides fractionated by RP-HPLC were lyophilized and re-suspended in 20% acetonitrile in 0.1% TFA prior to injection into the mass spectrometer source at a flow rate of 500 nL/min. Before performing a tandem mass spectrum, an ESI/MS mass spectrum (TOF MS mode) was acquired for each HPLC fraction over the mass range of 400–2000 *m/z*, in order to select the ion of interest, subsequently, these ions were fragmented in the collision cell (TOF MS/MS mode). Different collision energies were used, depending on the mass and charge state of the ions. The resulting ion spectra was acquired in the TOF analyser and deconvoluted using the MassLynx-MaxEnt 3 algorithm. Singly charged spectra were processed manually using the PepSeq application included in MassLynx.

### 2.11. Pharmacological Activity

#### 2.11.1. Young Chicken Biventer Cervicis Preparation

Male chicks (4–8-days-old) were killed with isoflurane and the biventer cervicis muscle was removed [22]. The biventer cervicis muscles were mounted under a tension of 0.5 g, in a 5 mL organ bath (Automatic organ multiple-bath LE01 Letica Scientific Instruments. Barcelona, Spain) at 37°C containing aerated (95% O_2_ - 5% CO_2_) Krebs solution (pH 7.5) of the following composition (mM): NaCl 118.7, KCl 4.7, CaCl_2_ 1.88, KH_2_PO_4_ 1.17, MgSO_4_ 1.17, NaHCO_3_ 25.0 and glucose 11.65. Contracture to exogenously applied acetylcholine (ACh; 55 and 110 *μ*M for 60 s) and KCl (20.1 mM for 130 s) was obtained in the absence of field stimulation, prior to the addition of a single dose of BrTX-I (50 *μ*g/mL). A bipolar platinum ring electrode was placed around the tendon, which runs the nerve trunk supplying the muscle. Indirect stimulation was performed with a (MAIN BOX LE 12404 Panlab s.l. Powerlab AD Instruments Barcelona, Spain) stimulator (0.1 Hz, 0.2 ms, 3-4 V). Muscle contractions and contractures were isometrically recorded by force-displacement transducers (Model MLT0201 Force transducer 5 mg–25 g Panlab s.l. AD Instruments Pty Ltd. Spain) connected to a PowerLab/4SP (OUAD Bridge AD Instruments, Barcelona, Spain).

#### 2.11.2. Myotoxic Activity

Groups of four Swiss mice (18–20 g) received an intramuscular (i.m.) or an intravenous (i.v.) injection of variable amounts of BrTX-I, in 100 *μ*L of PBS, in the gastrocnemius. A control group received 100 *μ*L of PBS. At different intervals of time (2, 4, 6, 9, and 24 h) blood was collected from the tail into heparinized capillary tubes, and the plasma creatine kinase (CK; EC 2.7.3.2) activity was determined by a kinetic assay (Sigma 47-UV). Activity was expressed in U/L, one unit defined as the phosphorylation of 1 *μ*mol of creatine/min at 25°C.

#### 2.11.3. Edema-Forming Activity

The ability of BrTX-I to induce edema was studied in groups of five Swiss mice (18–20 g) according Ponce-Soto et al. [6, 23, 24]. Twenty microliters of phosphate-buffered saline (PBS; 0.12 M NaCl, 0.04 M sodium phosphate, pH 7.2) with BrTX-I (1, 5, 10 and 20 *μ*g/paw) were injected in the subplantar region of the right footpad. The control group received an equal volume of PBS alone. The swelling of the paw was measured at 0.5; 1; 3; 6, and 24 h after administration. Edema was expressed as the percentage increased in the volume of the treated group to that of the control group at each time interval.

#### 2.11.4. Cytokines

The percentage of cytotoxicity was of IL-1, IL-6, and TNF-*α* in the plasma were collected and measured at 30, 60, 180, and 360 min after i.p. injection of the BrTX-I PLA_2_ (1.0 mg/kg) (20 *μ*g/100 *μ*L) or sterile saline. After centrifugation, the supernatants were used for determination of IL-1 and IL-6 levels by a specific EIA. The levels of cytokines IL-1, IL-6, and TNF-*α* in the serum from BALB/c mice were assayed by a two-site sandwich enzyme-like immunosorbent assay (ELISA). In brief, ELISA plates were coated with 100 *μ*L (1 *μ*g/mL) of the monoclonal antibodies anti-IL-1, in 0.1 M sodium carbonate buffer (pH 8.2) and incubated for 6 hours at room temperature. The wells were then washed with 0.1% phosphate-buffered saline (PBS/Tween-20) and blocked with 100 *μ*L of 10% fetal calf serum (FCS) in PBS for 2 hours at room temperature. After washing, duplicate sera samples of 50 *μ*L were added to each well. After 18 hours of incubation at 4°C, the wells were washed and incubated with 100 *μ*L (2 *μ*g/mL) of the biotinylated monoclonal antibodies anti-IL-1, anti-IL-6,as second antibodies for 45 minutes at room temperature. After a final wash, the reaction was developed by the addition of orthophenyldiamine (OPD) to each well. Optical densities were measured at 405 nm in a microplate reader were measured using a VersaMax 190 multiwell plate reader (Molecular Devices, Sunnyvale, CA, USA).

The cytokine content of each sample was read from a standard curve established with the appropriate recombinant cytokines (expressed in picograms per millilitre). The minimum levels of each cytokine detectable in the conditions of the assays were 10 pg/mL for IL-1, IL-6.

### 2.12. Statistical Analysis

The results are reported as the means ± SEM. The significance of differences among the means was assessed by ANOVA followed by Dunnett's test when various experimental groups were compared to the control group. A value of *P* < 0.05 indicated significance.

## 3. Results

**Figure 1:**
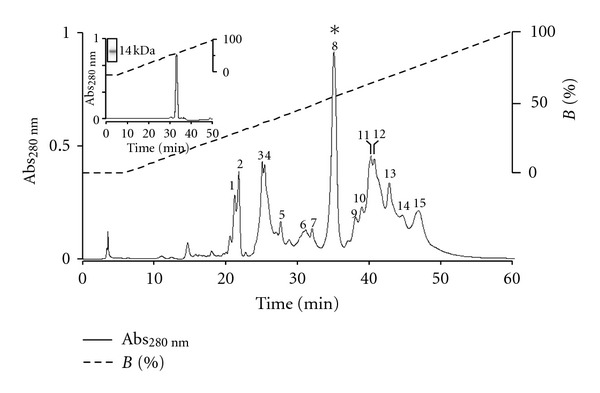
Elution profile of *Bothrops roedingeri* venom by RP-HPLC on an m-Bondapack C18 column. Fraction 4 (BrTX-I) contained PLA_2_ activity. Insert Re-chromatography on RP-HPLC chromatography of the BrTX-I and electrophoretic profile of BrTX-I with molecular mass ~14 kDa).

The elution profile of *Bothrops roendigeri *venom following RP-HPLC performed on a C18 column showed fifteen fractions (1–15) (Figure 1). The fifteen eluted peaks were screened for PLA_2_ activity. Only the fraction labeled in figure peak 8 presented PLA_2_ activity, which was eluted with 58% of buffer B. 

**Figure 2:**
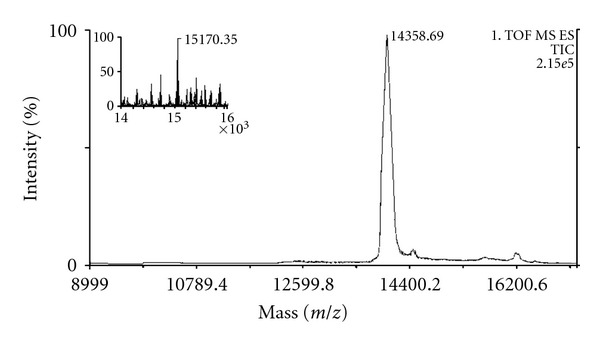
Mass determinations of BrTX-I by mass spectrometry, using a Q-Tof Ultima API ESI/MS (TOF MS mode). Insert mass spectrum, showing multiple alkylation channels of alkylated BrTX-I PLA_2_ isolated from *Bothrops roedingeri*.

To confirm the level of purity, peak 8 was re-purified in a *μ*-Bondapack C 18 column (0.78 cm × 30 cm; Waters 991-PDA system) in HPLC of the reverse phase, showing a high level of molecular homogeneity (95%), for the presence of a single peak for the peak 8 (BrTX-I, with a very small retention time difference (37.19 ± 0.34 min) (Figure 1 insert). SDS-PAGE show of PLA_2_ BrTX-I only band with molecular masses of ~14 kDa (Figure 1 insert) confirmed by MALDI-TOF mass spectrometry in 14,358.69 Da (Figure 2).

The amino acid composition determined was: N, D/10; Q, E/7; S/6; G/6; H/3; R/9; T/6; A/5; P/7; Y/8; V/5; M/1; C/14; I/5; L/7; F/3; K/18; W/Not determined (Figure 5(f)).

Samples of the native with mass 14,358.69 Da (Figure 2) and alkylated 15,170.35 Da (Figure 2 inserted) BrTX-I were digested with trypsin and the digests were analyzed by RP-HPLC.  Table 1 shows the masses of the tryptic peptides obtained for from the BrTX-I. It is possible to see that these proteins presented five common peptides to the other *Bothrops* snake venoms. The data obtained were processed using the Mascot MS/MS Ion Search software (http://www.matrixscience.com/).

To obtain detailed structural information, the native protein was alkylated and then digested to be analyzed through ESI-MS/MS. The alkylated protein digest was fractionated by RP-HPLC and each chromatographic peak marked in the chromatogram was manually collected and lyophilized. *De novo *sequencing by ESI-MS/MS was carried out for each peptide peak. The sequences were deduced using ESI-MS/MS and 5 peptides were obtained from the alkylated BrTX-I (Table 1).

Ile and Leu residues were not discriminated in any of the sequences, since they were indistinguishable in low-energy collision-induced dissociation spectra. Due to the external calibration applied to all the spectra, it was also not possible to distinguish between Gln and Lys residues based on the 0.035 Da that separates these amino acids, except for Lys, marked in bold in Table 1, which was deduced by analysis of the cleavage and missed cleavage sites of the enzyme.

Each *de novo *sequenced peptide of the BrTX-I was submitted separately to the NCBI database, using the protein search program BLAST-p with the search being restricted to the sequenced proteins from the PLA_2_ from snake venom family. In order to determine the presence and number of cysteine residues, BrTX-I was reduced and alkylated as described in Section  2.6.

The protein mass registered in peak 1–4 after alkylation was 15170.35 Da; the mass increase of 812 Da indicated the presence of 14 Cys modified residues. The primary structure of the BrTX-I was determined by sequence tryptic digested and deduction of the SwissProt database http://br.expasy.org/. BrTX-I presented a sequence of 54 amino acid residues sequenced, being BrtX-I: DLWQWNKMIK - - - - - - - - - - - - -YGCYCGW GGR- - - - - - - - - - - - - - - - - - - - - - -LTGC P- - - - - - - - - -KDITIVCGE DLPC- - - - - - -KAAAVCFYE NLGTYNKK- - - - - - - - - - - - -

From BrTX-I, five peptides, with molecular masses of 1,360.65 Da (peak 1), 1,404.67 Da (peak 2), 1,791.07 Da (peak 3), 1,120.28 Da (peak 4), and 616.79 Da (Peak 5). After the determination of these molecular masses and with the utilization of iodoacetamide, the cysteines presented in the peptides were alkylated (Table 1).

**Figure 3:**
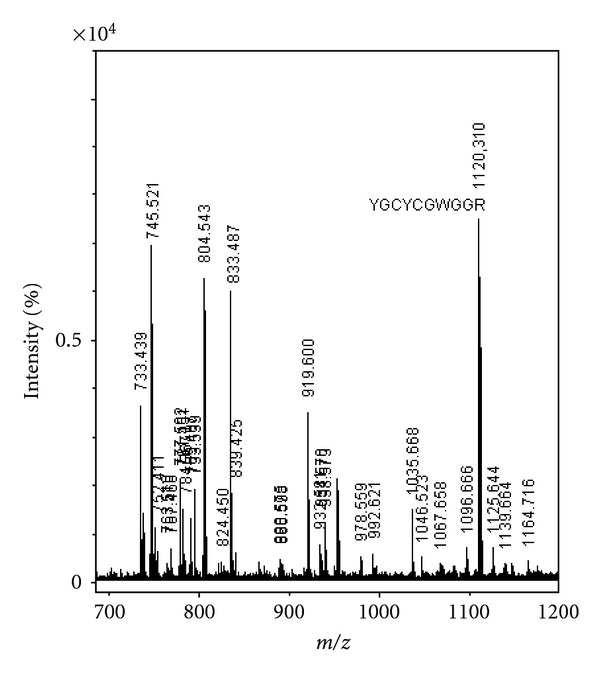
MS/MS spectrum of the peptide tryptic ion of *m/z* 1120.310. Ion of the major sequence-specific peptide of the complementing ions YGCYCGWGGR, from which the sequence of BrTX-I tag was deduced.

**Figure 4:**
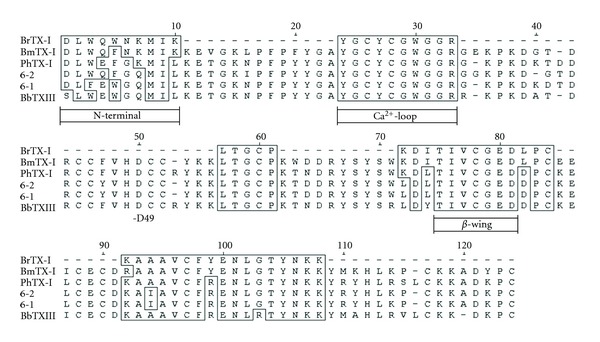
Alignment of the deduced amino acid sequence of the new PLA_2_ BrTX-I with PLA_2_ present in venom of PLA_2_ (BmTX-I) from *Bothrops moojeni *[26], PLA_2_ PhTX-I from *Porthidium hyoprora* [27], PLA_2_ isoforms (6-1 and 6-2) of the fraction BthTX-II from *Bothrops jararacuçu* [15], and BbTX-III from *Bothrops brazili* [27].

The peptide eluted in fraction 4 of BrTX-I, having the sequence Y G C Y C G W G G R (tandem MS spectra shown in Figure 3) and the sequence of the BrTX-I protein was deduced and returns high homology with the others PLA_2_s from snake from *Bothrops* snake genus present of the venoms snake registered in the date base Blast-p and showed high sequence homology with other PLA_2_ in the region associated with the catalytic site (Figure 4).

**Figure 5:**
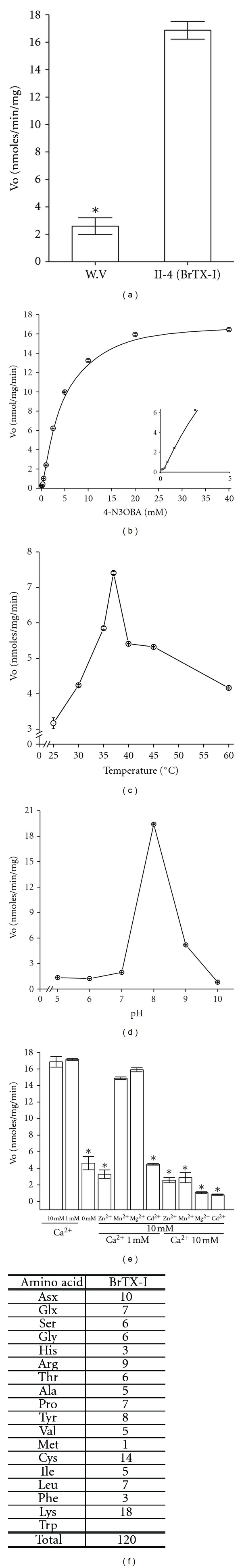
(a) PLA_2_ activity of *Bothrops roedingeri* venom and peak 4 (BrTX-I); (b) effect of substrate concentration on the kinetics of BrTX-I (PLA_2_) activity. (c) effect of temperature on the PLA_2_ activity of BrTX-I; (d) effect of pH on BrTX-I activity; (e) influence of ions (10 mM each) on PLA_2_ activity in the absence or presence of 1 mM Ca^2+^. The results of all experiments are the mean ± SE, of three determinations (*P* < 0.05) and (f) amino acid composition of BrTX-I from *Bothrops roendingeri* snake venom.

The PLA_2_ activity was examined in the *Bothrops roedingeri* venom and in BrTX-I using the synthetic substrate 4-nitro-3(octanoyloxy) benzoic acid [25]. The PLA_2_ activity was higher in BrTX-I c (Figure 5(a)). Under the conditions used, BrTX-I showed a discrete sigmoidal behavior (Figure 5(b) insert), mainly at low substrate concentrations. Maximum enzyme activity occurred at 35–40°C (Figure 5(c)) and the pH optimum was 8.0 (Figure 5(d)). PLA_2_s require Ca^+2^ for full activity, being only 1 mM of Ca^+2^ needed for BrTX-I to present phospholipase A_2_ activity. The addition of Zn^2+^, Mg^2+^, Mn^2+^, and Cd^2+^ (10 mM) in the presence of low Ca^2+^ concentration (1 mM) decreases the enzyme activity. The substitution of Ca^2+^ by Mg^2+^, Cd^2+^and Mn^2+^ also reduced the activity to levels similar to those in the absence of Ca^2+^ (Figure 5(e)).

**Figure 6:**
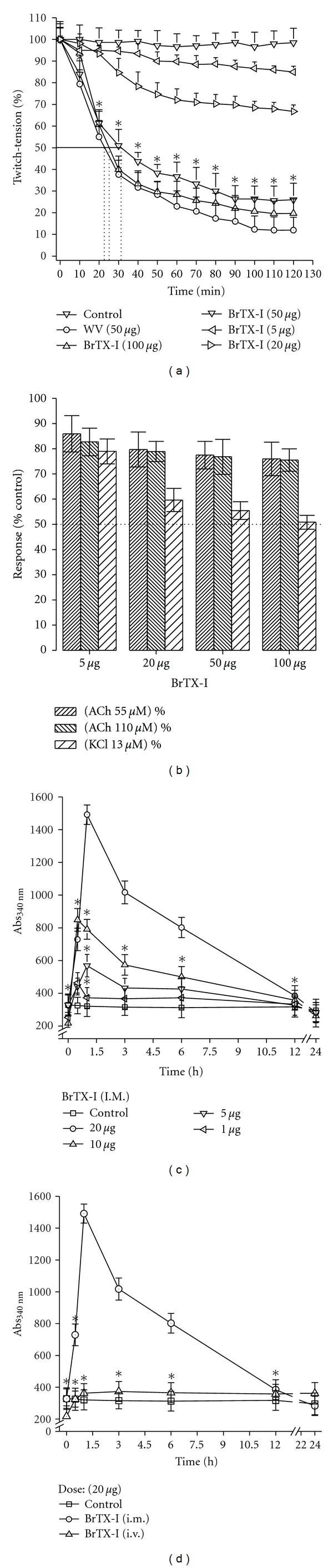
(a) Neuromuscular blockade in chick biventer cervicis muscle preparation (BCP), after addition of *B. roedingeri* whole venom (50 *μ*g/mL), or fraction BII-4 (BrTX-I; 5, 20, 50, and 100 *μ*g/mL). (b) Inhibition of the response to ACh and KCl, after a 120 min incubation with PLA_2_ BrTX-I of *Bothrops roedingeri* (5, 20, 50, and 100 *μ*g/mL) in chick biventer cervicis muscle preparation. Each point represents the average from five experiments ± SEM. *P* < 0.05 compared with control. In (c), a group of five Swiss mice (18–20 g) received an intramuscular (i.m.) injection of BrTX-I (1 to 20 *μ*g in 50 *μ*L of PBS), in the gastrocnemius muscle of mice. (d) CK serum levels after control (□) or PLA_2_ BrTX-I injection by the i.m. route (○) and i.v route (Δ). At different times, blood was collected, and serum CK levels were measure. Values are means ± SEM of five mice at each point.

In the neuromuscular activity in chick nerve-muscle preparation, the whole venom concentrations of the 50 *μ*g/mL were tested as well as the concentrations of 5, 20, 50, and 100 *μ*g/mL of BrTX-I. The tested concentration, in both venom and BrTX-I, caused an irreversible dose-dependent blockade of the neuromuscular transmission (*P* < 0.05). The time required for the venom to achieve 50% twitch tension blockade, through an indirect stimulation, was: 22.60 ± 0.61 min (50 *μ*g/mL) (Figure 6). The time required for BrTX-I to achieve 50% twitch tension blockade, also through indirect stimulation only doses of 50 (31.51 ± 0.52 min) and 100 *μ*g/mL (25.29 ± 0.28 min) (Figure 6(a)). The twitch tension records of the control preparation remain stable at 98% to the venom and 97% to the BrTX-I (5 *μ*g) along the 120 min of incubation with Krebs solution.

Regarding the venom, the concentration of 50 *μ*g/mL altered significantly the ACh (110 *μ*M) and KCl (20 mM) induced contractures when compared to the control values. In the concentration of the 50 *μ*g/mL, the complete blockade was not accompanied by significantly inhibition of the response to ACh and KCl (Figure 6(b)). In the control preparations, the contracture to ACh and KCl was kept stable after a 120 min indirect stimulation.

*In vivo, *BrTX-I induced a conspicuous local myotoxic effect when injected by the i.m. route, only doses 10 and 20 *μ*g (Figure 6(c)), but no increase in plasma CK levels occurred after their i.v. injection even in the same dose of 20 *μ*g. Time-course analysis showed a maximum increase in plasma CK 1 h after i.m. injection, returning to normal by 24 h (Figure 6(d)).

**Figure 7:**
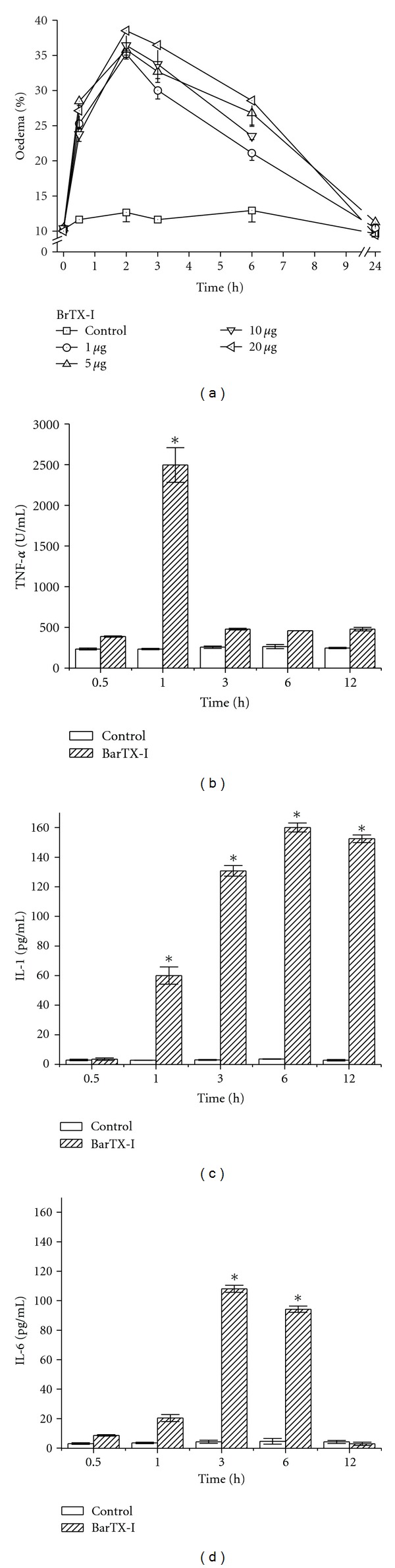
In (a), time-course of the mice paw oedema induced by selected doses of BrTX-I (1–20 *μ*g). The oedema, which was expressed as the percentage increased in the volume of the treated group to that of the control group at each time interval, was maximal around 2 h and decreased thereafter. Levels of TNF-*α*, IL-1 and IL-6 ((b), (c), and (d), resp.) in the serum after injection of BrTX-I. Animals were injected i.m. with BrTX-I (1.0 mg/kg) or sterile saline alone (control) in a final volume of 1 mL. TNF-*α*, IL-1 and IL-6 ((b), (c), and (d), resp.) were quantified by specific ELISA, in serum collected at the indicated time intervals after BrTX-I or saline injection as described in Section 2. Each bar represents mean GSEM of 5 animals. *P < 0.05 when compared with the corresponding control.

Compared to PBS-injected animals, those which received subplantar injections of the BrTX-I (1, 5, 10 and 20 *μ*g/paw) presented marked paw edema all doses (Figure 7(a)). Maximal activity was attained 2 h to BrTX-I after injection and receded to normal levels after 24 h. The level of edema induction by 20 *μ*g of BrTX-I PLA_2_ was similar to the other doses tested.

To further analyze and compare the mechanisms of the inflammatory events induced by BrTX-I PLA_2_, the concentrations of the IL-1, IL-6, and TNF-*α* in the serum were measured. BrTX-I caused a marked increase in the TNF-*α* concentrations only at 1 h (Figure 7(b)). In both the case of IL-1, the maximum peak was recorded at 6 h, on the other hand for IL-6 level the peak was at 3 h (Figures 7(c) and 7(d)).

## 4. Discussion

The purification procedure for basic PLA_2_s developed by Ponce-Soto et al. [6, 15, 23, 24] showed to be also efficient for the obtainment of the Bothropsroedingeritoxin I PLA_2_ (BrTX-I) from *Bothrops roedingeri* snake venom. Fractionation protocol of this crude venom using a single pass chromatographic in a column *μ*-Bondapack C-18 coupled to a system of reverse phase HPLC (0.78 cm–30 cm; Waters 991-PDA system) gave rise to 15 fractions at 280 nm, the eight last being the basic PLA_2_ named BrTX-I (Figure 1).

SDS-PAGE showed (Figure 1 insert) the isolated toxin, BrTX-I have Mr of ~14 kDa similarly to basic PLA_2_ isolated from other myotoxins from *Bothrops* snake venoms.

The molecular masses obtained by MALDI TOF mass spectrometry showed to be similar to that of other snake venom PLA_2_s (14358.69 Da) (Figure 2). Sequence homology studies had showed that there are extremely conserved positions in the PLA_2_s. In positions 1 and 2, there is a predominance of the amino acids sequence (DL), in position 4 (Q). One of the highly conserved regions in the amino acid sequences of PLA_2_ is the Ca^2+^-binding loop, segment from…YGCYCGXGG… and HD(49)CC (Figure 3). Residues forming the Ca^2+^-binding loop and the catalytic network of BrTX-I PLA_2_ show a high conservation grade, reflecting the nondecreased catalytic activity.

The primary structure of BrTX-I determined by deduced sequencing (SwissProt database http://br.expasy.org/) method is aligned with the sequences of some other homologous snake venom PLA_2_ from snake of the crotalidae family. It was very similar to that of other PLA_2_ (Figure 4).

The PLA_2_ activity showed to be higher in BrTX-I (16.87 ± 0.643 nmoles/min/mg) when compared with the whole venom (2.59 ± 0.617 nmoles/min/mg) (Figure 5(a)). The PLA_2_ from *Crotalus durissus terrificus* venom is a typical PLA_2_, since it hydrolyzes synthetic substrates at position 2 and preferentially attacks substrates in their micellar state [28]. They can hydrolyze phospholipids in monomeric, micellar or lipid bilayer phases. PLA_2_ enzymes exhibit a large and abrupt increase (up to 10,000 times) in their catalytic activity when monomeric phospholipids aggregate forms micelles at their critical micellar concentration [29]. This is due to the higher efficiency of interfacial catalysis, which depends on the absorption of the enzyme onto the lipid-water interface, strongly promoted by the presence of anionic amphipatic molecules within the membrane [30]. With synthetic substrate, BrTX-I behaved allosterically, especially at low substrate concentrations, which is in agreement with the results obtained by Beghini et al. [31], Bonfim et al. [32, 33], Ponce-Soto et al. [18], Calgarotto et al. [26], Huancahuire-Vega et al. [27] and for other PLA_2_ using the same nonmicellar substrate also observed that the dependence of activity on substrate concentration was markedly sigmoidal (Figure 5(b)).

The PLA_2_s from snake are highly stable and resistant to heat, acid, and urea, but catalytic activity is inactivated at high pH. When micellar substrates are used, maximum catalytic activity occurs at pH 7-8 and 30–55°C [17, 28, 34–36] (Figure 5(c)). BrTX-I showed maximum enzyme activity at 35–45°C and greatest activity at around pH 8.0 (Figure 5(d)).

A strict requirement for Ca^2+^ is characteristic of some PLA_2_ [18, 31, 35, 37]. BrTX-I showed typical Ca^2+^-dependent PLA_2_ activity similar to other PLA_2_ and this activity was lower in the presence of other divalent cations. Beghini et al. [31] observed the same for PLA_2_ from *Crotalus durissus cascavella* venom and Ponce-Soto et al. [18] for PLA_2_ from *Crotalus durissus collilineatus* (Figure 5(e)).

The amino acid composition of the BrTX-I PLA_2_ toxin revealed a high content of basic and hydrophobic residues, with 14 half-Cys, in agreement with the reported compositions and primary structures of PLA_2_ toxins isolated from *Bothrops *venoms (Figure 5(f)), [6, 15, 38, 39]. The pharmacological activities investigated for BrTX-I PLA_2_ includes neurotoxicity *ex vivo* in preparation BCP, *in vivo* inducing rapid damaging action to skeletal muscle tissue, paw oedema and increase of IL-1, IL-6 and TNF-*α* in the mice serum.

Some authors [3, 4, 6, 15, 40–44] have proposed several models to explain PLA_2 _catalytic and pharmacological activities. In these models PLA_2_ has two separated places; one is responsible for catalitic activity and other for biological activity expression. In according to them, the pharmacological place would be located in the surface of PLA_2_ molecules.

The BrTX-I caused an irreversible concentration-dependent blockade of the indirectly elicited twitch responses of the chick biventer cervicis muscle preparation (BCP). Only doses 20, 50, and 100 *μ*g/mL caused an irreversible dose-dependent blockade of the neuromuscular transmission (Figure 6(a)). The complete blockade of the muscle contraction all of the doses, was not accompanied by any significant inhibition of the responses to ACh. Inhibition response to KCl was progressive in terms of increasing the dose, suggesting a myotoxic effects due to destabilization of the membrane (Figure 6(b)).

Thus, the neuromuscular blockade produced by BrTX-I may be attributed to presynaptic activity, either by blocking axonal conduction or by affecting transmitter release at the motor nerve-terminal. The fact that the BrtX-I from* Bothrops moojeni* did not significantly affect the response to ACh and KCl, except when high doses were used, suggests that the venom presents a primordial presynaptic nature. Such neuromuscular blockade characteristics have been attributed to presynaptic-acting PLA_2_ from snake [45, 46] as those of *Crotalus durissus terrificus* [47], *Micrurus species* [48, 49], and other *Bothrops*, *Bothrops insularis* [50], *Bothrops pauloensis* [47, 51], and *Bothriopsis bilineata smargadina* [52], which did not show any detectable effect on the nicotinic receptor and, in some cases, showed only a mild muscle alteration.

In according to the model proposed by [42], the anticoagulant place would be located in a region between the 53 and 76 residues, considering this region charged positively in the PLA_2 _with high anti-coagulant activity. In PLA_2_ with moderate or low anti-coagulant activity, there is a predominancy of negative chargings. This region is placed in a distinct local and separated of foreseen regions by neurotoxicity and myotoxicity.

Local and systemic skeletal muscle degeneration is a common consequence of envenomations due to snakebites and mass bee attacks. PLA_2_ is an important myotoxic component in these venoms, inducing a similar pattern of degenerative events in muscle cells. The bothropics PLA_2_ myotoxins generally present low systemic toxicity, in contrast to myotoxic PLA_2_ that are also strongly neurotoxic [5, 53].

Our studies on local and systemic myotoxicity *in vivo* reveal the BrTX-I is nonsystemic myotoxin with local action due to decrease of the plasmatic CK levels (Figures 6(a) and 6(b)). This fact reinforces the hypothesis of differentiated action of local and systemic myotoxicity proposed by Gutiérrez and Ownby [5] and also the unspecificity and specificity proposed by Kini [3], Ponce-Soto et al. [6] and Gutiérrez et al. [54].

PLA_2_s from snake venoms exert a large number of pharmacological activities [35, 54] due to a process of accelerated micro-evolution through which a high mutational rate in the coding regions of their genes has allowed the development of new functions, mainly associated with the exposed regions of the molecules [13]. The integral analysis of the inflammation elicited by BrTX-I from *Bothrops roedingeri* venom in the mouse serum performed in the present study allowed a parallel evaluation of the increase in microvascular permeability, by paw oedema and the production of various inflammatory mediators.

The PLA_2_s from snake induced an increase in vascular permeability in peritoneal cavity of mice. This is in agreement with previous observations on the edema forming activity of similar molecules in the rodent footpad model [55, 56]. The increase of vascular permeability was detected after BrTX-I injection and developed rapidly, indicating that the observed plasma extravasation is primarily due to formation of endothelial gaps in vessels of microcirculation (Figure 7(a)). Previous studies have documented polymorphonuclear and mononuclear cellular infiltrate after injection of myotoxic PLA_2_s from the venoms of *Bothrops asper* [57], *Bothrops nummifer *[58], and *Bothrops jararacussu* [59] in mouse skeletal muscle, and after intrapleural administration of similar myotoxins from *Bothrops jararacussu* and *Bothrops pirajai* venoms [60]. The mediators involved in this effect of BrTX-I was not addressed in this study. However, the immediate plasma extravasation in response to BrTX-I, strongly suggests the involvement of vasoactive mediators derived from mast cell granules. Previously, the ability of venom PLA_2_ to degranulate mast cells has been shown [55].

TNF-*α* is also likely to be involved in inflammation induced by BrTX-I, since the PLA_2_ caused a significant increase of TNF-*α* levels in the serum. TNF-*α* is also likely to be involved in leukocyte infiltration induced by BrTX-I, since the PLA_2_ caused a significant increase of TNF-*α* levels in the serum. TNF-*α* induces the expression of E-selectin, CD11b/CD18 and ICAM-1 and triggers the release of several cytokines such as IL-1 and IL-6 (Figure 7(b)). Thus, our results suggest that TNF-*α* may have a role in the expression of CD18 and the release of other cytokines following BrTX-I injection, thereby being relevant for neutrophil influx and for increase of vascular permeability on the paw edema.

Cytokines, such as IL-1, IL-6, and TNF-*α*, are also relevant mediators for leukocyte migration and participate in several inflammatory conditions. Our results showed that BrTX-I induce increase in IL-1 and IL-6 in the serum, exerting a stronger effect (Figures 7(c) and 7(d)). IL-1 induced the expression of adhesion molecules by endothelial cells and stimulates the release of both IL-6 and TNF-*α* [61]. Thus, our results suggest that IL-1 may contribute for the leukocyte migration.

All these biological effects induced by the BrTX-I occur in the presence of a measurable PLA_2_ activity. Although the catalytic activity of PLA_2_ contributes to pharmacological effects, it is not a prerequisite [55, 56, 62–64]. However, further studies are necessary to identify the structural determinants involved in these pharmacological activities.

## Figures and Tables

**Table 1:** Measured molecular masses and deduced amino acid sequences obtained by ESI-MS/MS based on the alkylated tryptic peptides of BrTX-I. The peptides were separated by RP-HPLC and sequenced by mass spectrometry. C = alkylated cysteine, lysine residues shown in bold were deduced on the cleavage and missed cleavage by trypsin. All molecular masses are reported as monoisotopic.

BrTX-I HPLC fraction	Measured mass (Da)	Amino acid sequence	Theoretical mass (Da)
1	1360.65	DL/IWQ/KWNK/QMI/L**K/Q**	1360.61
2	1404.67	DI/LTI/LVCGEDL/IPC**K/Q**	1404.64
3	1791.07	AAAVCFYENL/IGTYNK/Q**K/Q**	1791.03
4	1120.28	YGCYCGWGGR	1020.25
5	616.79	L/ITGCP**K/Q**	616.75
